# Application of Next-Generation Sequencing Following Tandem Mass Spectrometry to Expand Newborn Screening for Inborn Errors of Metabolism: A Multicenter Study

**DOI:** 10.3389/fgene.2019.00086

**Published:** 2019-02-14

**Authors:** Yuqi Yang, Leilei Wang, Benjing Wang, Shuang Liu, Bin Yu, Ting Wang

**Affiliations:** ^1^Changzhou Maternity and Child Health Care Hospital Affiliated to Nanjing Medical University, Changzhou, China; ^2^Lianyungang Maternal and Child Health Hospital Affiliated to Yangzhou University, Lianyungang, China; ^3^The Affiliated Suzhou Hospital of Nanjing Medical University, Suzhou, China

**Keywords:** newborn screening, inborn errors of metabolism, tandem mass spectrometry, next-generation sequencing, gene diagnosis

## Abstract

This study explored the effectiveness of expanding newborn screening (NBS) by tandem mass spectrometry (TMS) and gene diagnosis by next-generation sequencing (NGS). First, we described the characteristics of gene variants in Jiangsu Province. We collected clinical data from three NBS centers. All infants followed a unified screening and diagnosis process. After obtaining informed consent, dried blood spots (DBSs) were collected and analyzed by TMS. If the results fell outside of the cut-off value, repeat analysis was performed. If the re-test results remained abnormal, the infant was recalled for further assessment. We performed targeted sequencing using the extended edition panel of inborn errors of metabolism (IEM) to detect 306 genes using the Illumina HiSeq 2500 platform. A total of 536,008 babies underwent NBS by TMS in three NBS centres. In total, 194 cases were eventually diagnosed with various types of inherited metabolic diseases, with an overall incidence of 1/2763. There were 23 types of diseases, including ten amino acid disorders (43.5%), eight organic acidaemias (34.8%) and five fatty acid oxidation defects (21.7%). In these infants, we clearly identified variants of disease-causing genes by next-generation sequencing (NGS). Most had two variants and others had one or three variants: 88% of gene variants were heterozygous and 12% were homozygous. There is a certain incidence of IEM in Jiangsu Province and it is necessary to carry out screening for 27 diseases. Meanwhile, NGS combined with TMS offers an enhanced plan for NBS for IEM.

## Introduction

Newborn screening (NBS) is an important public health program for improving children's health (Keskinkç, 2014; Berry, 2015) and is widely used throughout the world. It screens infants shortly after birth to identify conditions that are treatable but not clinically evident in the newborn period. The negative sequelae associated with a subset of genetic disorders can be averted or improved if the child is diagnosed early. Many countries carry out screening for diseases such as congenital hypothyroidism (CH), phenylketonuria (PKU), congenital adrenal hyperplasia (CAH), cystic fibrosis (CF), and hemoglobinopathy disorders (Wilcken and Wiley, 2008; Bhattacharya et al., 2014).

Since the 1990s, the application of tandem mass spectrometry (TMS) has allowed the expansion of NBS programmes (La Marca, 2014; Ombrone et al., 2016). Because of its sensitive, specific nature and its ability to analyse dozens of metabolites simultaneously, it can prompt diagnosis of more inborn errors of metabolism (IEM). NBS has advanced to one comprehensive and complex screening system from a simple blood or urine screening test and can detect over 50 different IEM diseases (Therrell et al., 2015). In 2006, the American College of Medical Genetics (ACMG) set out the guidelines of NBS program and confirmed 29 types of screening diseases [American College of Medical Genetics Newborn Screening Expert Group, 2006; Centers for Disease Control and Prevention (CDC), 2006]. Subsequently, the United Kingdom, Germany, Australia, South Korea, Japan, and other countries also applied TMS technology to expand neonatal screening (Bodamer et al., 2007; Downing and Pollitt, 2008). In China, NBS with TMS began in 2001 (Lin et al., 2001). Currently, an increasing number of NBS centers have carried out this work (Han et al., 2007; Sun et al., 2011; Guo et al., 2018; Yang et al., 2018).

It is well-known that IEM mostly follow an autosomal recessive inheritance pattern (i.e., the gene variants are mostly inherited from the parents). In addition, the clinical manifestations of IEM are complex and highly variable. It is also difficult for clinicians to interpret the screening results of IEM because it involves many indicators and ratios. Therefore, a further diagnosis of suspected positive children by TMS is very important. Detection of diseases-caused genes variant could contribute to IEM diagnosis. Next-generation sequencing (NGS) technology has gradually become of prime importance in the field of genetic diagnosis. It can sequence millions of DNA molecules simultaneously and be adapted to many aspects of biomedical research. In recent years, NGS has been increasingly and widely applied in clinical practice and has profoundly changed the diagnosis, prognosis and treatment of many human diseases (Xuan et al., 2013; Yohe and Thyagarajan, 2017). Since 2014, we have applied NGS as the second key step in NBS program. It can be used for the diagnosis of high-risk infants found by TMS. Thus, far, we (three neonatal disease screening centers from Jiangsu Province) have already carried out a total of 500,000 population clinical studies and confirmed the genetic diagnosis by NGS.

In the present study, we systematically reviewed the work of NBS program with TMS over the past 4 years and collected clinical data from three NBS centers. We explored the effectiveness of expanding NBS by TMS and gene diagnosis by NGS and preliminarily describe the characteristics of gene variants of IEM. We hope to improve the quality of NBS program.

## Materials and Methods

### Patients and Study Design

From January 2014 to June 2018, a total of 536,008 newborn infants who underwent NBS by TMS were recruited for this study. These infants came from three NBS centers in Jiangsu Province: Changzhou Maternity and Child Health Care Hospital affiliated with Nanjing Medical University, the Lianyungang Maternal and Child Health Hospital affiliated with Yangzhou University and the Affiliated Suzhou Hospital of Nanjing Medical University. Table 1 showed the baseline characteristics of all infants.

**Table 1:** Baseline characteristics of all infants.

	***n***	**Constituent ratio (%)**
Total	536,008	100.0
**SEX**		
Boy	282,261	52.7
Girl	253,729	47.3
Unknown	18	0.0
**NUMBER OF FETUSES**		
Single pregnancy	531,826	99.2
Twin pregnancies	4,153	0.8
Multiple pregnancies	29	0.0
**REGION**		
Urban	287,296	53.6
Suburban and rural	248,531	46.4
Unknown	181	0.0
**BIRTH WEIGHT**		
≥2,500 g	515,972	96.3
2,500 g> birth weight ≥2,000 g	13,702	2.6
2,000 g> birth weight ≥1,500 g	3,799	0.7
<1,500 g	1,539	0.3
Unknown	996	0.2
**GESTATIONAL WEEK**		
Full term (≥37 weeks)	504,984	94.2
Premature (<37 weeks)	26,107	4.9
Unknown	4,917	0.9

The study design and protocol were reviewed and approved by the ethics committee of the Changzhou Maternity and Child Health Care Hospital affiliated with Nanjing Medical University. Written informed contents were obtained from the newborns' parents before screening.

### Laboratory Methodology

**Figure 1:**
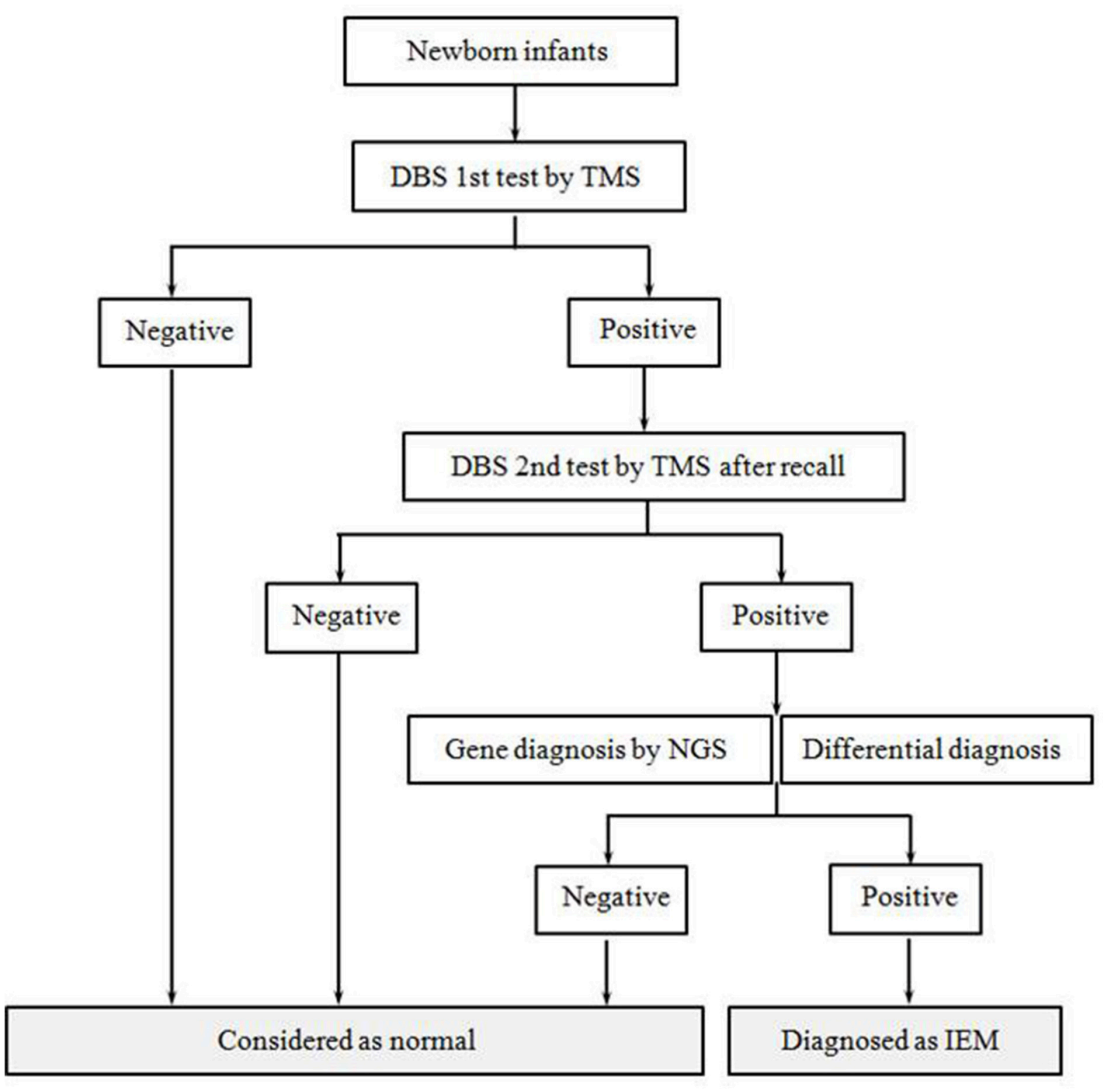
Flow diagram of newborn screening and the diagnostic process. After informed consent, all newborns were accepted screening and diagnosis according to this procedure. The procedure mainly included tandem mass spectrometry screening, positive recall test, differential diagnosis and gene diagnosis.

All infants followed a unified NBS and diagnosis process (Figure 1). The methods were as follows:

### NBS by TMS

After obtaining informed consent, dried blood spots (DBSs) were collected from all infants on 903 filter paper (Wallac Oy, Turku, Finland) at 72 h after birth. An unified experimental platform was used in all three centers. All DBSs were analyzed by using MS/MS with a NeoBase™ Non-derivatised MS/MS Kit (PerkinElmer, Turku, Finland). The lists of NBS disorders and the analytes included in the MS/MS screen were shown in Supplementary Tables 1, 2, respectively. The reference values were defined by determining 1,000 normal-term infants (0.5th percentile−99.5th percentile). If the results fell outside of the cut-off value, repeat analysis was performed. If the re-test results remained abnormal, the infant was recalled for further assessment. Based on the consideration of early diagnosis and intervention for positive cases, we adopted the strategy of diagnosis and treatment at the same time. Positive case diagnosis combines clinical manifestations, individualized assistant examination and gene detection, in which gene detection report is a very important basis. Regarding the positive cases after recall, individualized assistant examination were detected according to the disease type, mainly included routine blood tests, assessment of liver and kidney function, myocardial zymogram, blood gas, blood sugar, and blood ammonia. To some cases of amino acid or organic acid metabolic diseases, urine gas chromatography was used to supplemental diagnosis and differential diagnosis. Classically, target genes were detected by NGS at the same time.

### Gene Diagnosis by NGS Technology

#### DNA Extraction

Five milliliter of peripheral blood was taken from each subject and stored in an EDTA anticoagulant tube. Genomic DNA was extracted from 2 ml of peripheral whole blood using a Qiagen Blood DNA Mini Kit (Qiagen, Hilden, Germany) and preserved at −20°C after measuring the concentration. The remainder of the peripheral whole blood was preserved at −80°C.

### High-Throughput Sequencing

We performed targeted sequencing using the extended edition panel of inherited metabolic diseases (Genuine Diagnostic, Hanzhou, China) to detect 306 genes, including phenylalanine hydroxylase(*PAH*,MIM 612349), 6-pyruvoyltetrahydropterin synthase (*PTS*, MIM 612719), methylmalonyl-CoA mutase (*MUT*, MIM 609058), solute carrier family 22 member 5 (*SLC22A5*, MIM 603377) and so on (Supplementary Table 3). The target regions were enriched by multiple probe hybridization using an Agilent SureSelect Human Exon Sequence Capture Kit, and the capture products were purified using Agencourt AMPure XP Beads (Beckman Coulter, Brea, CA, USA). Purified products were treated with a TruePrep™ DNA Library Prep Kit V2 for Illumina (Vazyme Biotech Co., Ltd., Nanjing, China), and a special index was added using the TruePrep™ Index Kit V2 for Illumina (Vazyme). The quality of the DNA library was tested by Qubit and a 2100 Bioanalyser (Agilent High Sensitivity DNA Kit, Agilent Technologies, Santa Clara, CA, USA). Next, the sequencing libraries were quantified by using the Illumina DNA Standards and Primer Premix Kit (KAPA) and subjected to massively parallel sequencing on the Illumina HiSeq 2500 platform.

### Data Analysis

First, low-quality reads were filtered out from all sequencing data, and the remainder of the sequencing data were compared to the human genome reference sequence using the Burrows-Wheeler Aligner (BWA). Next, variants were called by Genome Analysis Toolkit (GATK) and annotated to public databases by Annovar. The effect of the variant on protein function was predicted according to the minor allele frequency (MAF) in a normal population, sequence conservation, change in the amino acid caused by the variant, and its position in the protein structure. After combining the clinical information, the pathogenicity of the variant was interpreted according to ACMG standards and guidelines.

#### Verification by Sanger Sequencing

Variants were confirmed by Sanger sequencing using specific primers. The polymerase chain reaction (PCR) conditions were according to TaKaRa LA PCR™ Kit Ver.2.1 (TaKaRa Bio Inc., Kusatsu, Japan). The PCR products were recovered and purified form agarose gels using a NucleoSpin® Gel and PCR Clean-up Kit (MACHEREY-NAGEL, Duren, Germany). All PCR products were diluted to 10 ng/μL for sequencing using the BigDye® Terminator v3.1 Cycle Sequencing Kit (Applied Biosystems Foster City, CA, USA) for amplification and purification. A total of 10 μL Hi-Di (Applied Biosystems) was added to each well. The DNA was then denatured at 95°C for 5 min, transferred to a 96-well plate after cooling, and sequenced on an ABI 3500XL (Applied Biosystems).

## Results

Over 4 years, 536,008 babies underwent NBS by TMS followed by NGS in three centers from Jiangsu Province, including 282,261 boys (52.7%) and 253,729 girls (47.3%). In the present population, the rate of premature infants and low-birth-weight infants was 4.9% and 3.6%, respectively. The baseline characteristics of all infants are shown in Table 1. After DBS screening by TMS, 17,764 babies received positive results. The positive rate of initial screening was 3.31%. A total of 13,865 cases (78.05%) were recalled successfully and received re-testing after re-sampling. After detection by TMS, 1033 cases still received positive results, and the positive rate of recall was 7.45%. These patients received a differential diagnosis and gene diagnosis. In total, 194 cases were diagnosed with various types of inherited metabolic diseases, with an overall incidence of 1/2763. Among them, the incidence of amino acid, organic acid, and fatty acid metabolism was 1/5414, 1/12465 and 1/10308, respectively. The parameters of the study project are shown in Table 2.

**Table 2:** Parameters of the study project.

**Project**	**Parameter**
Time for DBS collection (day)	4.0 (3~7)
Time for transport to lab (day)	3.5 (3~7)
Time for test (day)	2.0 (2~4)
Time for report (day)	3.5 (3~5)
Positive cases of initial screening	17,764
Positive rate of initial screening (%)	3.31
Positive recall cases	13,865
Positive recall rate of initial screening (%)	78.05
Positive cases after recall	1033
Positive rate of recall (%)	7.45
Confirmed cases	194
Incidence	1/2763
Amino acid metabolism	1/5414
Organic acid metabolism	1/12465
Fatty acid metabolism	1/10308
Positive predictive value (%)	1.40

**Figure 2:**
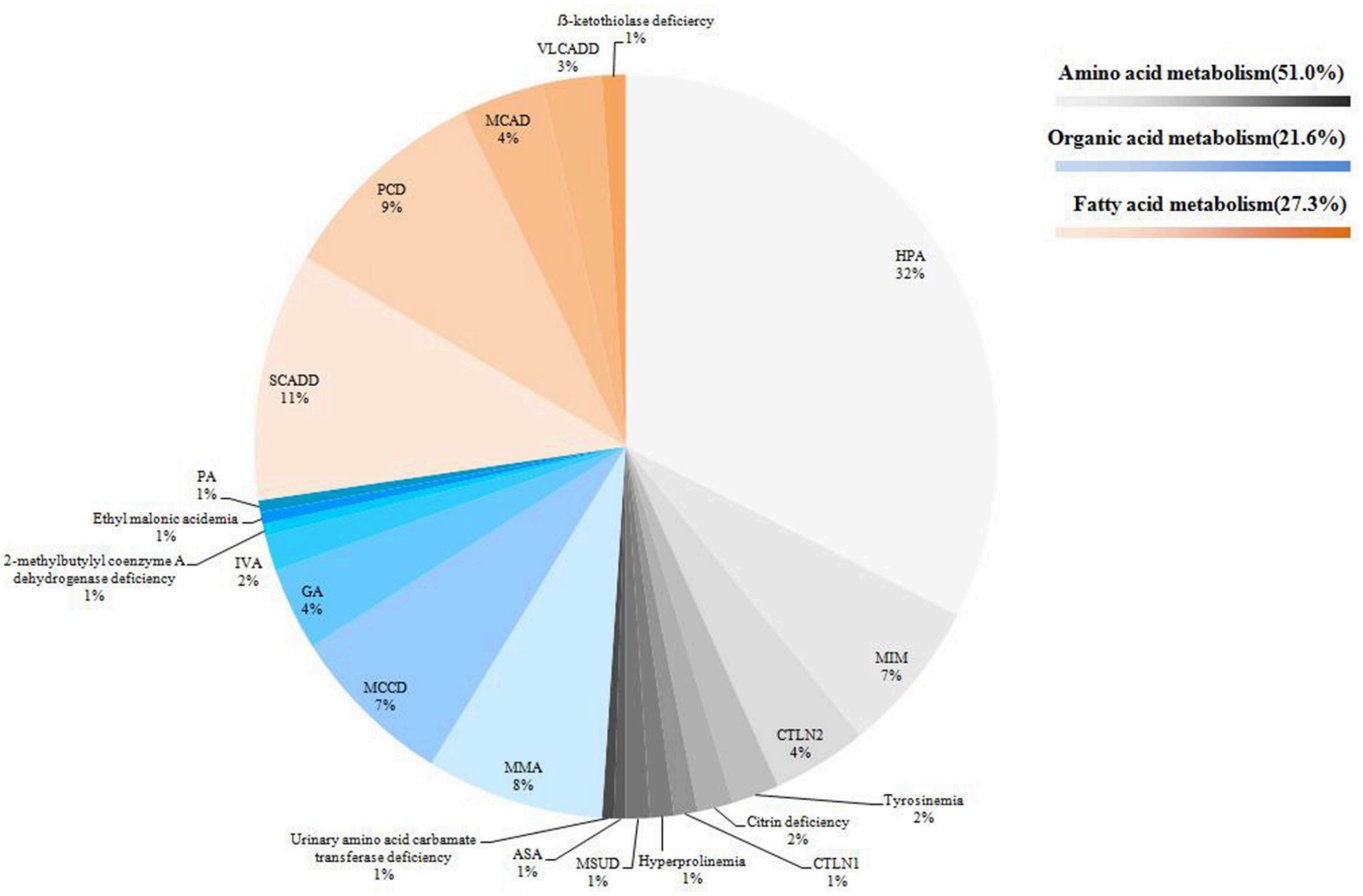
Distribution of all confirmed cases. According to the disease type, 194 cases were displayed by classification and propotion. Among them, amino acid metabolic diseases were expressed in gray system, organic acids in blue system and fatty acids in orange system.

In this study, we identified a total of 23 types of IEM, including 10 amino acid disorders (43.5%), eight organic acidaemias (34.8%) and five fatty acid oxidation defects (21.7%). The highest incidence was hyperphenylalaninaemia (HPA, 1/8508), followed by short chain acyl coenzyme A dehydrogenase deficiency (SCADD, 1/26800) and carnitine uptake defect (CUD, 1/29778). Some IEM were extremely rare. For some IEM diseases, we found only 1~2 cases in 536,008 newborns. The distribution of all confirmed cases is shown in Figure 2. In total, 95.4% (185/194) of cases followed up regularly, and the longest follow-up period was nearly 4 years. These children were treated or alimentary controlled according to the type and degree of disease, which mainly included: (1) drug therapy combined with diet control (e.g., glutaric acidemia I (GA-1), methylmalonic acidemia (MMA), and citrin deficiency; (2) drug therapy (i.e., babies with CUD were routinely supplemented with carnitine); (3) diet control (e.g., PKU); or (4) follow-up only (e.g., infants with 3-methylcrotonyl-CoA carboxylase deficiency (3MCCD), SCADD, and hypermethioninaemia (H-HET). Such patients often do not exhibit obvious clinical symptoms and do not receive special medication. These cases were also regularly tested for relevant indicators. Four cases died because of parental abandonment (one case was tyrosinaemia, one was maple syrup urine disease, and two cases were methylmalonic acidemia). Most infants died 1–2 months after birth. Five cases gave up treatment. Thus, far, the growth and development of the children in follow-up treatment is good (Table 3).

**Table 3:** Results of 194 infants with IEM.

**IEM**	**Phenotype MIM number**	***n***	**Incidence**	**Follow-up**
Amino acid metabolism		99	1/5414	
Hyperphenylalaninaemia (HPA)	261600/261640	63	1/8508	All cases followed up regularly
Hypermethioninaemia (H-HET)	250850/236200	13	1/41231	Two cases gave up treatment and the others followed up regularly
Citrullinaemia type II (CTLN2)	605814	8	1/67001	All cases followed up regularly
Tyrosinaemia	276700/276710/276600	4	1/134002	One case died and the others followed up regularly
Citrin deficiency	603471/605814	3	1/178669	All cases followed up regularly
Citrullinaemia type I (CTLN1)	215700	2	1/268004	All cases followed up regularly
Hyperprolinaemia	239500	2	1/268004	All cases followed up regularly
Maple syrup urine disease (MSUD)	248600	2	1/268004	One case died and the others followed up regularly
Argininosuccinic aciduria (ASA)	207900	1	1/536008	The case followed up regularly
Ornithine transcarbamylase deficiency (OTCD)	311250	1	1/536008	The case followed up regularly
Organic acid metabolism		43	1/12465	
Methylmalonic acidemia (MMA)	251000/277400	15	1/35734	Two cases died and the others followed up regularly
3-Methylcrotonyl-CoA carboxylase deficiency (3MCCD)	210200/210210	15	1/35734	One case gave up treatment and the others followed up regularly
Glutaric acidemia I (GA-1)	231670	6	1/89335	One case died, one case gave up treatment and the others followed up regularly
Isovaleric acidemia (IVA)	243500	3	1/178669	All cases followed up regularly
Glutaric acidemia I (GA-2)	231680	1	1/536008	The case followed up regularly
2-Methylbutylyl coenzyme A dehydrogenase deficiency	610006	1	1/536008	The case followed up regularly
Ethylmalonic encephalopathy	602473	1	1/536008	The case followed up regularly
Propionic acidemia (PA)	606054	1	1/536008	The case followed up regularly
Fatty acid metabolism		52	1/10308	
Short chain acyl coenzyme A dehydrogenase deficiency (SCADD)	201470	20	1/26800	One case gave up treatment and the others followed up regularly
Carnitine uptake defect (CUD)	212140	18	1/29778	All cases followed up regularly
Medium chain acyl coenzyme A dehydrogenase deficiency (MCAD)	201450	7	1/76573	All cases followed up regularly
Very long chain acyl coenzyme A dehydrogenase deficiency (VLCADD)	201475	5	1/107202	All cases followed up regularly
ß-Ketothiolase deficiency	203750	2	1/268004	All cases followed up regularly
Total		194	1/2763	

Except for HPA, 96.2% (126/131) of cases accepted genetic detection by NGS, while only 41.3% (26/63) of HPA infants received NGS testing because they could be diagnosed clearly by specific biochemical markers. Among the other IEM babies, we clearly identified variants of disease-causing genes. Their initial screening, re-testing and genetic testing results are shown in Supplementary Table 4. At three NBS centers, we screened 27 types of IEM using TMS followed by NGS: 26 diseases have an autosomal recessive inheritance pattern and only one (hypermethioninaemia) has an autosomal dominant inheritance pattern. Most of these cases had two variants in disease-causing genes, while the others had one or three variants. Among them, 88% (111/126) of gene variants were heterozygous and 12% were homozygous. Based on the ACMG classification, 50.9% of variants were pathogenic, 22.3% were probably pathogenic and 8.9% were of uncertain significance; 17.9% of variants have not been reported. In this study, 23 children's parents received the detection and comparison of corresponding disease-causing genes. All of the variants came from their parents; no *de novo* variant was identified.

To visualize the results of NGS and Sanger sequencing, we will use Glutaric academia I(GA-1) as an example. According to some reports, the incidence of GA-I is extremely low. Six cases (cases 114–119 in Supplementary Table 4) had complex heterozygous variant sites of the *GCDH* gene and were thus diagnosed with GA-I, with an incidence of 1/89335 (GCDH, MIM 608801). Eleven types of *GCDH* variants were found, including seven pathogenic and two probably pathogenic. For example, case 114 was identified as a compound heterozygote for two known variants: c.1235C>A (inherited paternally) and c.1244-2A>C (inherited maternally). Cases 115 and 116 also harbor two variants: c.892G>A and c.261_506-433delinsATA for case 115 and c.109_110delCA and c.416C>G for case 116. However, c.261_506-433delinsATA and c.109_110delCA are novel variants that have not been reported in the literature.

## Discussion

Newborn screening (NBS) is an important public health program for improving children's health. One of the most important innovations made was the application of TMS. TMS makes possible the simultaneous measurement of several metabolites and, consequently, the detection of several diseases in one blood spot and in a unique analysis (Villoria et al., 2016; Wagner et al., 2016). Three NBS centers have routinely used NBS for 27 types of inherited metabolic diseases, including amino acid, organic acid, and fatty acid metabolism diseases. It has been proven that there is a considerable incidence of amino acid metabolic diseases and that TMS is a very effective method for clinical screening.

With the application of TMS, an increasing number of inherited metabolic diseases have been discovered and recognized. Although their incidence varies greatly in different countries, these diseases are not rare. According to some studies around the world, the total incidence rates of IEM range from 1:13,205 to 1:1178. For example, the incidence of IEM is 1:2512 in Slovenia (Smon et al., 2018), 1:2700 in Australia (Estrella et al., 2014), 1:2916 in Malaysia (Yunus et al., 2016), 1:3065 in Singapore (Lim et al., 2014), 1:4300 in the USA (Frazier et al., 2006), 1:8557 in Japan, 1:13,205 in South Korea, and 1:2200 in Germany (Shibata et al., 2018). Even in China, the incidence varies greatly among regions. The incidence of IEM is 1:1178 in Jining (Guo et al., 2018), 1:5626 in Zhejiang (Huang et al., 2011), and 1:7030 in Taiwan (Shibata et al., 2018). However, there is no large-scale study from China. The subjects of our study were collected from three centers in Jiangsu Province located in southern and northern Jiangsu. Therefore, this study can represent the situation of IEM in Jiangsu Province. According to the 500,000 population studies, the overall incidence of IEM is 1/2763, which is relatively higher compared with previous reports, especially with respect to amino acid metabolism diseases. Therefore, it is very important to expanded NBS by TMS in Jiangsu Province.

It is well-known that inherited metabolic diseases encompass a large group of rare genetic diseases, and their spectrum and incidence differ greatly among populations. How many diseases should be screened? There is no uniform standard in the world. Different countries use different screening strategies. For example, in 2006, the ACMG updated the guidelines for NBS and recommended a uniform panel of 29 disorders in the USA (American College of Medical Genetics Newborn Screening Expert Group, 2006). Revealing the local disease spectrum will be useful for planning NBS programmes. In the present study, all three types of IEM in Jiangsu Province had a certain incidence. Amino acid metabolism was the highest, followed by fatty acid metabolism, while organic acid metabolism was slightly lower. The top five incidences of diseases were as follows (in order): HPA, SCADD, CUD, MMA, and MCCD. These results are consistent with those of Han (Han et al., 2015); they reported that HPA, citrin deficiency, MMA, and MADD were the most common inherited disorders in China (Han et al., 2015). Therefore, in China, most NBS centers that already perform NBS by TMS screen 27 types of diseases. However, we believe that the diseases identified and strategies used should be adjusted with the accumulation of clinical data, including disease spectrum, ability of diagnosis, and treatment. For example, we identified 21 infants with SCADD. Until now, the longest follow-up period was 3 years. However, there were no clinical symptoms in these cases. Therefore, it is worth determining whether SCADD still needs to be included in routine screening.

Most IEM have no specific clinical manifestations, and their clinical manifestations are complex and highly variable. Some confirmed cases only show a slightly abnormal concentration of analytes or even negative screening results. However, metabolic disorders can occur rapidly under stress. At the same time, TMS screening involves many indicators and ratios; therefore, it is difficult to judge. Thus, it is very important to diagnose cases with a suspicious positive result accurately after TMS. The detection of disease-causing gene variants could contribute to a diagnosis of IEM. Sanger sequencing technology is time-consuming and laborious because it needs to establish libraries for every variant of every disease-causing gene. Its diagnostic efficiency is very low and is not suitable for clinical practice. In recent years, NGS technology has gradually played a key role in the field of gene diagnosis. It can simultaneously carry out large-scale parallel sequencing of millions of DNA molecules. It was also recently proven to be valuable in explaining abnormal metabolite concentrations in NBS, as it enables the differentiation between affected patients and mere heterozygotes and has improved the turnaround time of genetic analyses (Qian et al., 2017; Smon et al., 2018). In this study, we designed and established a panel for IEM gene diagnosis based on NGS, which included 306 IEM disease-related genes. According to our results, all cases with a disease-causing gene were identified using this NGS panel. Meanwhile, this technology would greatly shorten the diagnosis time, avoid a misdiagnosis, promote early treatment, and improve prognosis.

The molecule of interest is ionized into charged particles with different mass-charge ratios by TMS. A certain mass of ions is selected by first-order mass spectrometry (MS1) to enter the collision chamber, to produce sub-ions or neutral molecules, and is then detected by second-order mass spectrometry (MS2). The parent ions are paired with sub-ions or neutral molecules for analysis. DBSs are extracted with methanol, which contains amino acids and acylcarnitines as internal standards. Following derivatisation with n-butyl alcohol hydrochloric acid, samples are tested using MS/MS (Han et al., 2015). This method can simultaneously detect more than 50 amino acids and acylcarnitines in a single drop of blood and, thereby, carry out a rapid screening and diagnosis of more than 40 metabolic disorders in amino acids, organic acids, and fatty acid oxidation (Han et al., 2007). MS/MS can be helpful in facilitating the early diagnosis and timely treatment of inherited metabolic disorders. However, this method can only detect the condition of the blood at that time, not continuous changes. NGS is a revolutionary change to traditional sequencing that aims to sequence hundreds of thousands to millions of DNA molecules simultaneously. The DNA is fragmented and sequenced. There are overlapping areas between fragments, and the complete genome sequence can be obtained by sequence comparison. NGS can detect the cause of disease at the genetic level but is time-consuming and costly. At the same time, NGS technology can be used as an effective method for gene diagnosis after TMS screening. It can contribute to detect variations of diseases-caused genes conveniently and accurately, so as to help the diagnosis of IEM. However, at present, it is not possible to provide individualized treatment for different variations of genes. So NGS technology is not helpful to guide the treatment of children.

## Conclusion

We expanded NBS with TMS and screened 27 types of IEM diseases. We designed and established a panel of IEM disease-related genes followed by TMS for a clear diagnosis. Based on research from a population of 500,000, we confirmed a certain incidence of IEM in Jiangsu Province and determined that it is necessary to carry out screening for 27 diseases. Meanwhile, NGS combined with TMS is an enhanced plan for NBS detecting IEM.

## Author Contributions

BY, LW, and TW carried out the assays and participated in the study design. YY, BW, and SL carried out clinical consultations, laboratory tests and performed the statistical analysis. BY conceived the study, participated in its design and coordination and helped draft the manuscript.

### Conflict of Interest Statement

The authors declare that the research was conducted in the absence of any commercial or financial relationships that could be construed as a potential conflict of interest.
