# Supplementary material for: Application of Next-Generation Sequencing Following Tandem Mass Spectrometry to Expand Newborn Screening for Inborn Errors of Metabolism: A Multicenter Study
Source: Front Genet. 2019 Feb 14;10:86. doi: 10.3389/fgene.2019.00086 (PMC6382741; doi:10.3389/fgene.2019.00086)
Supplement: Supplementary file 1 [file Table_1.DOC]

**Supplementary table 1. The list of NBS disorders included in the MS/MS screen**

|  | **Disease** | **Disease code** |
| --- | --- | --- |
| **Amino acid metabolic disorders** | | |
| 1 | Phenylketonuria | PKU |
| 2 | Tetrahydrobiopterin deficiency | BH4D |
| 3 | Maple syrup urine disease | MSUD |
| 4 | Citrullinemia type Ι | CTLN1 |
| 5 | Citrin deficiency (citrullinemia type II) | NICCD |
| 6 | Tyrosinemia | HT |
| 7 | Argininemia | ARG1 |
| 8 | Hypermethioninemia | H-MET |
| 9 | Ornithine transcarbamylase deficiency | OTCD |
| 10 | Argininosuccinic aciduria | ASA |
| **Organic acid metabolic disorders** | | |
| 11 | Methylmalonic acidemia | MMA |
| 12 | Propionic acidemia | PA |
| 13 | Isovaleric acidemia | IVA |
| 14 | Holocarboxylase synthetase deficiency | HCS |
| 15 | Glutaric acidemia type I | GA-Ι |
| 16 | Beta-ketothiolase deficiency | BKT |
| 17 | 3-Methylcrotonyl-CoA carboxylase deficiency | 3MCC |
| 18 | 3-Hydroxy-3-Methylglutaryl-coenzyme a lyase deficiency | HMGCL |
| 19 | Isobutyrylglycinuria | IBG |
| **Fatty acid metabolic disorders** | | |
| 20 | Carnitine uptake defect | CUD |
| 21 | Carnitine palmitoyltransferase I deficiency | CPT-Ι |
| 22 | Short-chain acyl-CoA dehydrogenase deficiency | SCAD |
| 23 | Glutaric acidemia type II | GA-II |
| 24 | Medium-chain acyl-CoA dehydrogenase deficiency | MCAD |
| 25 | Very long-chain acyl-CoA dehydrogenase deficiency | VLCAD |
| 26 | Carnitine Palmitoyltransferase II Deficiency | CPT-II |
| 27 | Carnitine Acylcarnitine Translocase Deficiency | CACT |
