# Supplementary material for: Application of Next-Generation Sequencing Following Tandem Mass Spectrometry to Expand Newborn Screening for Inborn Errors of Metabolism: A Multicenter Study
Source: Front Genet. 2019 Feb 14;10:86. doi: 10.3389/fgene.2019.00086 (PMC6382741; doi:10.3389/fgene.2019.00086)
Supplement: Supplementary file 2 [file Table_2.DOC]

**Supplementary table 2. The list of the analytes included in the MS/MS screen**

|  | **Analyte Name** | **Short name** |
| --- | --- | --- |
| 1 | Alanine | Ala |
| 2 | Arginine | Arg |
| 3 | Citrulline | Cit |
| 4 | Glycine | Gly |
| 5 | Leucine/Isoleucine/Hydroxyproline | Leu/lle/Pro-OH |
| 6 | Methionine | MET |
| 7 | Ornithine | Orn |
| 8 | Phenylalanine | Phe |
| 9 | Proline | Pro |
| 10 | Tyrosine | Tyr |
| 11 | Valine | Val |
| 12 | Carnitine free | C0 |
| 13 | Acetylcarnitine | C2 |
| 14 | Propionylcarnitine | C3 |
| 15 | Malonylcarnitine +3-Hydroxybutyrylcarnitine | C3DC + C4OH |
| 16 | Butyrylcarnitine+Isobutyrylcarnitine | C4 |
| 17 | Methylmalonylcarnitine +3-Hydroxyisovalerylcarnitine | C4DC + C5OH |
| 18 | Isovalerylcarnitine+Methylbutyrylcarnitine | C5 |
| 19 | Tiglylcarnitine | C5:1 |
| 20 | Glutarylcarnitine +3-Hydroxyhexanoylcarnitine | C5DC + C6OH |
| 21 | Hexanoylcarnitine | C6 |
| 22 | Methylglutarylcarnitine | C6DC |
| 23 | Octanoylcarnitine | C8 |
| 24 | Octenoylcarnitine | C8:1 |
| 25 | Decanoylcarnitine | C10 |
| 26 | Decenoylcarnitine | C10:1 |
| 27 | Decadienoylcarnitine | C10:2 |
| 28 | Dodecanoylcarnitine | C12 |
| 29 | Dodecenoylcarnitine | C12:1 |
| 30 | Tetradecanoylcarnitine | C14 |
| 31 | Tetradecenoylcarnitine | C14:1 |
| 32 | Tetradecadienoylcarnitine | C14:2 |
| 33 | 3-Hydroxytetradecanoylcarnitine | C14OH |
| 34 | Palmitoylcarnitine | C16 |
| 35 | Palmitoleylcarnitine | C16:1 |
| 36 | 3-Hydroxypalmitoylcarnitine | C16OH |
| 37 | 3-Hydroxypalmitoleylcarnitine | C16:1OH |
| 38 | Stearoylcarnitine | C18 |
| 39 | Oleoylcarnitine | C18:1 |
| 40 | Linoleoylcarnitine | C18:2 |
| 41 | 3-Hydroxystearoylcarnitine | C18OH |
| 42 | 3-Hydroxyoleoylcarnitine | C18:1OH |
| 43 | Succinylacetone | SA |
