# Supplementary material for: Application of Next-Generation Sequencing Following Tandem Mass Spectrometry to Expand Newborn Screening for Inborn Errors of Metabolism: A Multicenter Study
Source: Front Genet. 2019 Feb 14;10:86. doi: 10.3389/fgene.2019.00086 (PMC6382741; doi:10.3389/fgene.2019.00086)
Supplement: Supplementary file 3 [file Table_3.DOC]

**Supplementary table 3. The list of 306 genes in the panel**

| **Gene** | **Gene Annotation（OMIM）** |
| --- | --- |
| *PAH* | [Hyperphenylalaninemia, non-PKU mild], 261600 (3), Autosomal recessive; Phenylketonuria, 261600 (3), Autosomal recessive |
| *PTS* | Hyperphenylalaninemia, BH4-deficient, A, 261640 (3), Autosomal recessive |
| *PCBD1* | Hyperphenylalaninemia, BH4-deficient, D, 264070 (3), Autosomal recessive |
| *QDPR* | Hyperphenylalaninemia, BH4-deficient, C, 261630 (3), Autosomal recessive |
| *SPR* | Dystonia, dopa-responsive, due to sepiapterin reductase deficiency, 612716 (3), ?Autosomal dominant, Autosomal recessive |
| *GCH1* | Dystonia, DOPA-responsive, with or without hyperphenylalaninemia, 128230 (3), Autosomal recessive, Autosomal dominant; Hyperphenylalaninemia, BH4-deficient, B, 233910 (3), Autosomal recessive |
| *BCKDHA* | Maple syrup urine disease, type Ia, 248600 (3), Autosomal recessive |
| *BCKDHB* | Maple syrup urine disease, type Ib, 248600 (3), Autosomal recessive |
| *DBT* | Maple syrup urine disease, type II, 248600 (3), Autosomal recessive |
| *DLD* | Dihydrolipoamide dehydrogenase deficiency, 246900 (3), Autosomal recessive |
| *AMT* | Glycine encephalopathy, 605899 (3), Autosomal recessive |
| *GCSH* | Glycine encephalopathy, 605899 (3), Autosomal recessive |
| *GLDC* | Glycine encephalopathy, 605899 (3), Autosomal recessive |
| *MAT1A* | Hypermethioninemia, persistent, autosomal dominant, due to methionine adenosyltransferase I/III deficiency, 250850 (3), Autosomal recessive, Autosomal dominant; Methionine adenosyltransferase deficiency, autosomal recessive, 250850 (3), Autosomal recessive, Autosomal dominant |
| *CBS* | Homocystinuria, B6-responsive and nonresponsive types, 236200 (3), Autosomal recessive; Thrombosis, hyperhomocysteinemic, 236200 (3), Autosomal recessive |
| *CTH* | Cystathioninuria, 219500 (3), Autosomal recessive; Homocysteine, total plasma, elevated, 0 (3) |
| *MTHFR* | Homocystinuria due to MTHFR deficiency, 236250 (3), Autosomal recessive; {Neural tube defects, susceptibility to}, 601634 (3), Autosomal recessive; {Schizophrenia, susceptibility to}, 181500 (3), Autosomal dominant; {Thromboembolism, susceptibility to}, 188050 (3), Autosomal dominant; {Vascular disease, susceptibility to}, 0 (3) |
| *SUOX* | Sulfite oxidase deficiency, 272300 (3), Autosomal recessive |
| *MOCS1* | Molybdenum cofactor deficiency A, 252150 (3), Autosomal recessive |
| *MOCS2* | Molybdenum cofactor deficiency B, 252160 (3), Autosomal recessive |
| *GPHN* | Molybdenum cofactor deficiency C, 615501 (3) |
| *FAH* | Tyrosinemia, type I, 276700 (3), Autosomal recessive |
| *TAT* | Tyrosinemia, type II, 276600 (3), Autosomal recessive |
| *HPD* | Hawkinsinuria, 140350 (3), Autosomal dominant; Tyrosinemia, type III, 276710 (3), Autosomal recessive |
| *HGD* | Alkaptonuria, 203500 (3), Autosomal recessive |
| *MUT* | Methylmalonic aciduria, mut(0) type, 251000 (3), Autosomal recessive |
| *MMAA* | Methylmalonic aciduria, vitamin B12-responsive, 251100 (3), Autosomal recessive |
| *MMAB* | Methylmalonic aciduria, vitamin B12-responsive, due to defect in synthesis of adenosylcobalamin, cblB complementation type, 251110 (3), Autosomal recessive |
| *MMACHC* | Methylmalonic aciduria and homocystinuria, cblC type, 277400 (3), Autosomal recessive |
| *MMADHC* | Homocystinuria, cblD type, variant 1, 277410 (3), Autosomal recessive; Methylmalonic aciduria and homocystinuria, cblD type, 277410 (3), Autosomal recessive; Methylmalonic aciduria, cblD type, variant 2, 277410 (3), Autosomal recessive |
| *LMBRD1* | Methylmalonic aciduria and homocystinuria, cblF type, 277380 (3), Autosomal recessive |
| *ABCD4* | Methylmalonic aciduria and homocystinuria, cblJ type, 614857 (3), Autosomal recessive |
| *MLYCD* | Malonyl-CoA decarboxylase deficiency, 248360 (3), Autosomal recessive |
| *CD320* | Methylmalonic aciduria due to transcobalamin receptor defect, 613646 (3) |
| *SUCLA2* | Mitochondrial DNA depletion syndrome 5 (encephalomyopathic with or without methylmalonic aciduria), 612073 (3), Autosomal recessive |
| *SUCLG1* | Mitochondrial DNA depletion syndrome 9 (encephalomyopathic type with methylmalonic aciduria), 245400 (3), Autosomal recessive |
| *SUCLG2* | 0 |
| *MCEE* | Methylmalonyl-CoA epimerase deficiency, 251120 (3), Autosomal recessive |
| *PCCA* | Propionicacidemia, 606054 (3), Autosomal recessive |
| *PCCB* | Propionicacidemia, 606054 (3), Autosomal recessive |
| *BTD* | Biotinidase deficiency, 253260 (3), Autosomal recessive |
| *GCDH* | Glutaricaciduria, type I, 231670 (3), Autosomal recessive |
| *IVD* | Isovaleric acidemia, 243500 (3), Autosomal recessive |
| *ACADSB* | 2-methylbutyrylglycinuria, 610006 (3), Autosomal recessive |
| *AUH* | 3-methylglutaconic aciduria, type I, 250950 (3), Autosomal recessive |
| *DNAJC19* | 3-methylglutaconic aciduria, type V, 610198 (3), Autosomal recessive |
| *CLPB* | 3-methylglutaconic aciduria, type VII, with cataracts, neurologic involvement and neutropenia, 616271 (3), Autosomal recessive |
| *TMEM70* | Mitochondrial complex V (ATP synthase) deficiency, nuclear type 2, 614052 (3), Autosomal recessive |
| *SERAC1* | 3-methylglutaconic aciduria with deafness, encephalopathy, and Leigh-like syndrome, 614739 (3), Autosomal recessive |
| *HMGCL* | HMG-CoA lyase deficiency, 246450 (3), Autosomal recessive |
| *MCCC1* | 3-Methylcrotonyl-CoA carboxylase 1 deficiency, 210200 (3), Autosomal recessive |
| *MCCC2* | 3-Methylcrotonyl-CoA carboxylase 2 deficiency, 210210 (3), Autosomal recessive |
| *HLCS* | Holocarboxylase synthetase deficiency, 253270 (3), Autosomal recessive |
| *ACADS* | Acyl-CoA dehydrogenase, short-chain, deficiency of, 201470 (3), Autosomal recessive |
| *ACADM* | Acyl-CoA dehydrogenase, medium chain, deficiency of, 201450 (3), Autosomal recessive |
| *ACADVL* | VLCAD deficiency, 201475 (3), Autosomal recessive |
| *HADH* | 3-hydroxyacyl-CoA dehydrogenase deficiency, 231530 (3), Autosomal recessive; Hyperinsulinemic hypoglycemia, familial, 4, 609975 (3), Autosomal recessive |
| *HADHA* | Fatty liver, acute, of pregnancy, 609016 (3); HELLP syndrome, maternal, of pregnancy, 609016 (3); LCHAD deficiency, 609016 (3); Trifunctional protein deficiency, 609015 (3), Autosomal recessive |
| *HADHB* | Trifunctional protein deficiency, 609015 (3), Autosomal recessive |
| *ACAD8* | Isobutyryl-CoA dehydrogenase deficiency, 611283 (3) |
| *ETHE1* | Ethylmalonic encephalopathy, 602473 (3), Autosomal recessive |
| *ETFA* | Glutaric acidemia IIA, 231680 (3), Autosomal recessive |
| *ETFB* | Glutaric acidemia IIB, 231680 (3), Autosomal recessive |
| *ETFDH* | Glutaric acidemia IIC, 231680 (3), Autosomal recessive |
| *ACAT1* | Alpha-methylacetoacetic aciduria, 203750 (3), Autosomal recessive|BETA-KETOTHIOLASE DEFICIENCY |
| *SLC22A5* | Carnitine deficiency, systemic primary, 212140 (3), Autosomal recessive |
| *SLC25A20* | Carnitine-acylcarnitine translocase deficiency, 212138 (3), Autosomal recessive |
| *CPT1A* | CPT deficiency, hepatic, type IA, 255120 (3), Autosomal recessive |
| *CPT2* | CPT II deficiency, lethal neonatal, 608836 (3), Autosomal recessive; CPT deficiency, hepatic, type II, 600649 (3), Autosomal recessive; {Encephalopathy, acute, infection-induced, 4, susceptibility to}, 614212 (3), Autosomal recessive, Autosomal dominant; Myopathy due to CPT II deficiency, 255110 (3), Autosomal recessive |
| *ARG1* | Argininemia, 207800 (3), Autosomal recessive |
| *ASL* | Argininosuccinic aciduria, 207900 (3), Autosomal recessive |
| *ASS1* | Citrullinemia, 215700 (3), Autosomal recessive |
| *SLC25A13* | Citrullinemia, adult-onset type II, 603471 (3), Autosomal recessive; Citrullinemia, type II, neonatal-onset, 605814 (3), Autosomal recessive |
| *CPS1* | Carbamoylphosphate synthetase I deficiency, 237300 (3), Autosomal recessive; {Pulmonary hypertension, neonatal, susceptibility to}, 615371 (3); {Venoocclusive disease after bone marrow transplantation}, 0 (3) |
| *OAT* | Gyrate atrophy of choroid and retina with or without ornithinemia, 258870 (3), Autosomal recessive |
| *OTC* | Ornithine transcarbamylase deficiency, 311250 (3), X-linked recessive |
| *SLC25A15* | Hyperornithinemia-hyperammonemia-homocitrullinemia syndrome, 238970 (3), Autosomal recessive |
| *G6PD* | Favism, 134700 (3), Autosomal dominant; Hemolytic anemia due to G6PD deficiency, 300908 (3); {Resistance to malaria due to G6PD deficiency}, 611162 (3) |
| *SLC5A5* | Thyroid dyshormonogenesis 1, 274400 (3), Autosomal recessive |
| *TG* | {Autoimmune thyroid disease, susceptibility to, 3}, 608175 (3); Thyroid dyshormonogenesis 3, 274700 (3), Autosomal recessive |
| *TPO* | Thyroid dyshormonogenesis 2A, 274500 (3), Autosomal recessive |
| *TSHB* | Hypothryoidism, congenital, nongoitrous 4, 275100 (3), Autosomal recessive |
| *TSHR* | Hyperthyroidism, familial gestational, 603373 (3); Hyperthyroidism, nonautoimmune, 609152 (3), Autosomal dominant, Isolated cases; Hypothyroidism, congenital, nongoitrous, 1, 275200 (3), Autosomal recessive; Thyroid adenoma, hyperfunctioning, somatic, 0 (3); Thyroid carcinoma with thyrotoxicosis, 0 (3) |
| *PAX8* | Hypothyroidism, congenital, due to thyroid dysgenesis or hypoplasia, 218700 (3), Autosomal recessive |
| *DUOX2* | Thryoid dyshormonogenesis 6, 607200 (3), Autosomal recessive |
| *CYP11B1* | Adrenal hyperplasia, congenital, due to 11-beta-hydroxylase deficiency, 202010 (3), Autosomal recessive; Aldosteronism, glucocorticoid-remediable, 103900 (3), Autosomal dominant |
| *CYP11B2* | Aldosterone to renin ratio raised, 0 (3); Hypoaldosteronism, congenital, due to CMO I deficiency, 203400 (3), Autosomal recessive; Hypoaldosteronism, congenital, due to CMO II deficiency, 610600 (3), Autosomal recessive; {Low renin hypertension, susceptibility to}, 0 (3) |
| *HSD3B2* | 3-beta-hydroxysteroid dehydrogenase, type II, deficiency, 201810 (3) |
| *STAR* | Lipoid adrenal hyperplasia, 201710 (3), Autosomal recessive |
| *CYP17A1* | 17-alpha-hydroxylase/17,20-lyase deficiency, 202110 (3), Autosomal recessive; 17,20-lyase deficiency, isolated, 202110 (3), Autosomal recessive |
| *CYP11A1* | Adrenal insufficiency, congenital, with 46XY sex reversal, partial or complete, 613743 (3) |
| *POR* | Antley-Bixler syndrome with genital anomalies and disordered steroidogenesis, 201750 (3), Autosomal recessive; Disordered steroidogenesis due to cytochrome P450 oxidoreductase, 613571 (3) |
| *ATP7B* | Wilson disease, 277900 (3), Autosomal recessive |
| *PC* | Pyruvate carboxylase deficiency, 266150 (3), Autosomal recessive |
