# Supplementary material for: Application of Next-Generation Sequencing Following Tandem Mass Spectrometry to Expand Newborn Screening for Inborn Errors of Metabolism: A Multicenter Study
Source: Front Genet. 2019 Feb 14;10:86. doi: 10.3389/fgene.2019.00086 (PMC6382741; doi:10.3389/fgene.2019.00086)
Supplement: Supplementary file 4 [file Table_4.doc]

**Supplementary table 4. The results of initial screening, re-testing and genetic testing**

| **IEM** | **n** | **Case** | **NBS result** | **Recall result** | **Gene** | **Gene MIM number** | **Nucleotide change** | **Genetic variant** | **Genetic mode** | **Variant type** | **Genetic source** |
| --- | --- | --- | --- | --- | --- | --- | --- | --- | --- | --- | --- |
| **Amino acid metabolism** | **99** |  |  |  |  |  |  |  |  |  |  |
| Hyperphenylalaninemia | 63 | 1 | phe:378.08 phr/tyr=5.78 | phe:458.85 phr/tyr=7.67 | *PAH* | 612349 | c.721c>t c.1068c>a | het | AR | P  LP | F  M |
|  |  | 2 | phe:829.36 phr/tyr=23.8 | phe:1691.91 phr/tyr=38.18 | *PAH* | 612349 | c.1068c>a | hom | AR | P |  |
|  |  | 3 | phe=1224.83,phe/tyr=19.60 | phe=1184.59 | *PAH* | 612349 | c.728g>a c.472c>t | het | AR | P  P | F M |
|  |  | 4 | phe=1309.83,phe/tyr=14.04 | phe=2302.74,phe/tyr=20.33 | *PAH* | 612349 | c.728g>a  - | het | AR | P |  |
|  |  | 5 | phe=345.84,phe/tyr=4.27 | phe=300.69 | *PAH* | 612349 | c.320a>g  - | het | AR | LP |  |
|  |  | 6 | phe=666.53,phe/tyr=5.82 | phe=712.69 | *PAH* | 612349 | c.1252a>c c.9137a>g | het | AR | LP P |  |
|  |  | 7 | phe=1261.91,phe/tyr=25.87 | phe=1475.60 | *PAH* | 612349 | c.442-1g>a c.755g>a | het | AR | P P |  |
|  |  | 8 | phe/tyr=2.43,phe=184.25 | phe/tyr=2.52,phe=293.68 | *PAH* | 612349 | c.310g>t  c.1238g>c | het | AR | US  P |  |
|  |  | 9 | phe/tyr=1.591,phe=138.77 | phe/tyr=1.516,phe=111.0 2 | *PAH* | 612349 | c.158g>a  c.1197a>t | het | AR | -  P |  |
|  |  | 10 | phe/tyr=1.59,phe=115 .59 | phe/tyr=2.14,phe=166.55 | *PAH* | 612349 | c.158g>a  c.728g>a(p.r243q) | het | AR | -  P |  |
|  |  | 11 | phe/tyr=1.53,phe=258.55 | phe/tyr=2.27,phe=258.35 | *PAH* | 612349 | c.1068c>a  c.1315+6t>a | het | AR | P  LP |  |
|  |  | 12 | phe/tyr=1.3,phe=158.61 | phe/tyr=1.94,phe=228.53 | *PAH* | 612349 | c.722g>a  c.1315+6t>a | het | AR | P  LP |  |
|  |  | 13 | phe/tyr=1.78,phe=176.22 | phe/tyr=1.85,p he=161.4 | *PAH* | 612349 | c.782g>a  c.464g>a | het | AR | LP  P |  |
|  |  | 14 | phe/tyr=1.547,phe=150.8 | leu+ile+pro-oh/phe=1.74,phe/(c3+c16)=79.07, phe=137.59（ | *PAH* | 612349 | c.158g>a  c.977g>a | het | AR | -  P |  |
|  |  | 15 | phe/tyr=2.27,phe=173.8 | phe/tyr=2.57,phe=212.6 | *PAH* | 612349 | c.1174t>a  c.728g>a | het | AR | -  P |  |
|  |  | 16 | phe=333.74,phe/tyr=4.48 | phe=333.74,phe/tyr=4.48 | *PAH* | 612349 | c.331c>t  c.1315+6t>a | het | AR | P  LP |  |
|  |  | 17 | phe=176.41 | phe=299.15,phe/tyr=2.05 | *PAH* | 612349 | c.208_210deltct  c.721c>t | het | AR | -  P |  |
|  |  | 18 | phe/tyr=4.56,phe=399.16 | - | *PAH* | 612349 | c.611a>g  c.721c>t | het | AR | P  P |  |
|  |  | 19 | phe/tyr=11.35,phe=647.1 | - | *PAH* | 612349 | c.125a>t  c.740g>t | het | AR | -  LP |  |
|  |  | 20 | phe/tyr=5.46,phe=365.84 | - | *PAH* | 612349 | c.721c>t  c.728g>a | het | AR | P  P |  |
|  |  | 21 | phe/tyr=9.06,phe=672.1 | - | *PAH* | 612349 | c.331c>t | hom | AR | P |  |
|  |  | 22 | phe=1768.16,phe/tyr=23.32 | - | *PAH* | 612349 | c.827t>g | hom | AR | - |  |
|  |  | 23 | phe/tyr=12.97,phe=974.66 | phe/tyr=14.39,phe=1117.64 | *PTS* | 612349 | c.155a>g  c.272a>g | het | AR | P  - |  |
|  |  | 24 | phe/tyr=3.28,phe=247.6 | phe/tyr=4.31,ph e=342.6 | *PTS* | 612349 | c.166g>a  c.259c>t | het | AR | LP  P |  |
|  |  | 25 | phe/tyr=2.04,phe=192.1 | phe/tyr=4.67,phe=307.2 | *PTS* | 612349 | c.166g>a  c.277c>a | het | AR | P  - |  |
|  |  | 26 | phe/tyr=16.51,phe=1211.54 | phe/tyr=14.94,phe=1271.58 | *PTS* | 612349 | c.259c>t | hom | AR | P |  |
|  |  | 27 | phe:1545.85 phr/tyr=34.82 | phe:1765.68 phr/tyr=31.38 | ND |  |  |  |  |  |  |
|  |  | 28 | phe:188.09 phr/tyr=2.57 | phe:132.29 phr/tyr=3.04 | ND |  |  |  |  |  |  |
|  |  | 29 | phe=459.26,phe/tyr=6.30 | phe=655.82 | ND |  |  |  |  |  |  |
|  |  | 30 | phe=449.22,phe/tyr=6.88 | phe=719.97,phe/tyr=11.44 | ND |  |  |  |  |  |  |
|  |  | 31 | phe=1262.23,phe/tyr=14.73 | phe=2084.31,phe/tyr=33.36 | ND |  |  |  |  |  |  |
|  |  | 32 | phe=790.97,phe/tyr=32.32 | phe=1272.89,phe/tyr=34.74 | ND |  |  |  |  |  |  |
|  |  | 33 | phe=449.67,phe/tyr=4.86 | phe=570.05,phe/tyr=7.07 | ND |  |  |  |  |  |  |
|  |  | 34 | phe=687.22,phe/tyr=9.28 | phe=922.63 | ND |  |  |  |  |  |  |
|  |  | 35 | phe=490.43,phe/tyr=11.01 | phe=1219.08 | ND |  |  |  |  |  |  |
|  |  | 36 | phe=422.06,phe/tyr=6.22 | phe=1623.31,phe/tyr=16.38 | ND |  |  |  |  |  |  |
|  |  | 37 | phe=452.18,phe/tyr=7.56 | phe=1386.18,phe/tyr=31.79 | ND |  |  |  |  |  |  |
|  |  | 38 | phe=536.21，phe/tyr=6.34 | phe=896.18，phe/tyr=10.06 | ND |  |  |  |  |  |  |
|  |  | 39 | phe=2330.68,phe/tyr=43.17 | phe=2318.89,phe/tyr=26.76 | ND |  |  |  |  |  |  |
|  |  | 40 | phe=754.73,phe/tyr=19.19 | phe=1369.79,phe/tyr=23.92 | ND |  |  |  |  |  |  |
|  |  | 41 | phe=1197.18，phe/tyr=14.31 | phe=1429.8，phe/tyr=14.03 | ND |  |  |  |  |  |  |
|  |  | 42 | phe=1847.46，phe/tyr=36.65 | phe=1681.9 | ND |  |  |  |  |  |  |
|  |  | 43 | phe=579.67,phe/tyr=8.87 | phe=932.16,phe/tyr=12.08 | ND |  |  |  |  |  |  |
|  |  | 44 | phe=533.38,phe/tyr=8.21 | phe=2048.53 | ND |  |  |  |  |  |  |
|  |  | 45 | phe=313.09,phe/tyr=3.08 | phe=441.05 | ND |  |  |  |  |  |  |
|  |  | 46 | phe=569.44,phe/tyr=5.44 | phe=711.23,phe/tyr=6.86 | ND |  |  |  |  |  |  |
|  |  | 47 | phe/tyr=2.01,phe=217.66 | phe/tyr=2.87,phe=280.84 | ND |  |  |  |  |  |  |
|  |  | 48 | phe/ tyr=1.72,phe=107.95 | phe/tyr=1.68,phe=155.32 | ND |  |  |  |  |  |  |
|  |  | 49 | leu+ile+pro-oh/phe=1.12,met/phe=0.11,cit/phe=0.09,phe=125 .09 | phe/tyr=1.11,p he=139.75 | ND |  |  |  |  |  |  |
|  |  | 50 | phe/tyr=9.6,phe=511.09 | - | ND |  |  |  |  |  |  |
|  |  | 51 | phe/tyr=11.96,phe=545.03 | - | ND |  |  |  |  |  |  |
|  |  | 52 | phe/tyr=9.31phe=441.82 | - | ND |  |  |  |  |  |  |
|  |  | 53 | phe/tyr=23.73,phe=973.99 | phe/tyr=37.19,phe=2038.9 | ND |  |  |  |  |  |  |
|  |  | 54 | phe/tyr=9.28,phe=686.67 | - | ND |  |  |  |  |  |  |
|  |  | 55 | phe/tyr=13.03,phe=629.2 | - | ND |  |  |  |  |  |  |
|  |  | 56 | phe/tyr=4.9,phe=401.32\| | - | ND |  |  |  |  |  |  |
|  |  | 57 | phe/tyr=9.58,phe=675.04 | - | ND |  |  |  |  |  |  |
|  |  | 58 | phe/tyr=13.33,phe=598.9 | - | ND |  |  |  |  |  |  |
|  |  | 59 | phe/tyr=4.27,phe=410.6 | - | ND |  |  |  |  |  |  |
|  |  | 60 | phe/tyr=29.92,phe=1766.96 | - | ND |  |  |  |  |  |  |
|  |  | 61 | phe/tyr=8.76,phe=760 | - | ND |  |  |  |  |  |  |
|  |  | 62 | phe/tyr=7.94,phe=549 | - | ND |  |  |  |  |  |  |
|  |  | 63 | phe/tyr=3.52,phe=399 | - | ND |  |  |  |  |  |  |
| Hypermethioninemia | 13 | 64 | met=1.1.1 | met=152.13 | *MAT1A* | 610550 | c.791g>a | het | AD | P | F |
|  |  | 65 | met=73.95 | met=75.19 | *CBS* | 613381 | c.215a>t | het | AD | US P |  |
|  |  | 66 | met=426.83 | met=428.69 | *MAT1A MCCC1* | 610550/609010 | c.550-1g>a; c.610g>a c.639+2t>a | het | AD | LP US P |  |
|  |  | 67 | met=73.62,met/phe=1.09 | met=98.94,met/phe=2.32 | *MAT1A* | 610550 | c.791g>a | het | AD | P |  |
|  |  | 68 | met/phe=1.52,met=64.81 | met/phe=6.05,met=252.23 | *MAT1A* | 610550 | c.791g>a | het | AD | P |  |
|  |  | 69 | met/phe=3.7,met=176.92 | met/phe=2.5,met=93.39 | *MAT1A* | 610550 | c.791g>a | het | AD | P |  |
|  |  | 70 | met/phe=2.2,met=87.91 | met/phe=5.36,met=269.99 | *MAT1A* | 610550 | c.791g>a | het | AD | P |  |
|  |  | 71 | met/phe=3.5,met=126.89 | met/phe=6.31,met=209.49 | *MAT1A* | 610550 | c.791g>a | het | AD | P |  |
|  |  | 72 | met=73.46,met/phe=1.45 | met=185.12,met/phe=5.27 | *MAT1A* | 610550 | c.791g>a | het | AD | P |  |
|  |  | 73 | met=109.45,met/phe=2.47 | met=246.17,met/phe=5.15 | *MAT1A* | 610550 | c.791g>a | het | AD | P |  |
|  |  | 74 | met=107.86,met/phe=2.811 | met=160.86,met/phe=3.935 | *MAT1A* | 610550 | c.791g>a | het | AD | P |  |
|  |  | 75 | met=70.2,met/phe=1.23 | met=70.2,met/phe=1.27 | *MAT1A* | 610550 | c.533c>t | het | AD | - |  |
|  |  | 76 | met=86.27,met/phe=1.8 | met=160.98,met/phe=2.92 | *MAT1A* | 610550 | c.776g>t | het | AD | P |  |
| Citrullinemia type Ι | 2 | 77 | cit=299.58 | cit=401.5 | *ASS1* | 603470 | c.1168g>a c.1128_1134delinsg | het | AR | P  LP | M  F |
|  |  | 78 | cit=197.42 | cit/arg=22.83,cit/phe=5.05,c18:1=0.31,orn/phe= 1.45,orn/cit=0.29,tyr/cit=0.45,(c0+c2+c3+c16+c18:1+c18)/c it=0.12,cit=249.35 | ND |  |  |  |  |  |  |
| Citrullinemia type II | 8 | 79 | cit=26.28 | cit=56.05;cit=274.29 | *SLC25A13* | 603859 | c.1638_1660dup c.615+5g>a | het | AR | -  P |  |
|  |  | 80 | ala/cit=7.6,cit=43.29 | cit=119.88,ala/cit=3.477 | *SLC25A13* | 603859 | c.1399c>t  c.852_855deltatg | het | AR | -  P |  |
|  |  | 81 | cit=38.08,ala/cit=6.787 | cit=476.19,ala/cit=0.39 | *SLC25A13* | 603859 | ivs16ins3kb  - | het | AR | - |  |
|  |  | 82 | cit=54.02,ala/cit=6.193 | cit=280.08,ala/cit=0.945 | *SLC25A13* | 603859 | c.852_855deltatg  - | het | AR | P |  |
|  |  | 83 | cit=68.83,ala/cit=5.54 | cit=71.65,ala/cit =3.51 | *SLC25A13* | 603859 | c.852_855deltatg  ivs16ins3kb | het | AR | P  - |  |
|  |  | 84 | cit=236.2 | - | *SLC25A13* | 603859 | c.1078c>t  ivs16ins3kb | het | AR | P  - |  |
|  |  | 85 | cit=534.92 | - | *SLC25A13* | 603859 | c.851_854delgtat | het | AR | P |  |
|  |  | 86 | cit=75.88 ala/cit=3 | cit=189.6 | ND |  |  |  |  |  |  |
| Tyrosinemia | 4 | 87 | sa=2.99,sa/phe=0.027 | sa=3.01,sa/phe=0.026 | *FAH* | 603859 | c.1210g>a  - | het | AR | LP |  |
|  |  | 88 | sa=11.36,sa/phe=0.069 | sa=11.6,sa/p he=0.093 | *FAH* | 603859 | c.1162g>a  - | het | AR | - |  |
|  |  | 89 | tyr=384.39,phe/tyr=0.13,leu+ile+pro-oh/tyr=0.48 | tyr=701.91,phe/tyr=0.08,leu+ile+pro-oh/tyr=0.39 | *HPD* | 609695 | c.784g>a  c.5c>t | het | AR | -  US |  |
|  |  | 90 | tyr=679.83,phe/tyr=0.069,leu+ile+pro-oh/tyr=0.169 | tyr=1034.16,phe/tyr=0.034,leu+ile+pro-oh/tyr=0.188 | *TAT* | 613018 | c.916c>t  c.236g>a | het | AR | -  US |  |
| Citrin deficiency | 3 | 91 | cit=120.61 | cit=371.54 | *SLC25A13* | 603859 | c.550c>t c.1638_1660d | het | AR | P P | F M |
|  |  | 92 | cit=255.32 | cit=518.51 | *SLC25A13* | 603859 | c.852_855del c.1638_1660d | het | AR | P P | F M |
|  |  | 93 | cit=53.28 | cit=370.45 | *SLC25A13* | 603859 | c.852_855deltatg  ivs16ins3kb | het | AR | P  P | M  F |
| Hyperprolinemia | 2 | 94 | pro=529.31 | pro=843.55 | *PRODH* | 606810 | c.1357c>t c.1414g>a | het | AR | -  - |  |
|  |  | 95 | pro=514.43 | pro=816.88 | *PRODH* | 606810 | c.1562a>g c.1414g>a | het | AR | P  - |  |
| Maple syrup urine disease | 2 | 96 | leu+ile+pro-oh=3110.88,val=716.61 | -- | *BCKDHB* | 248611 | c.1028delc | hom | AR | LP | F M |
|  |  | 97 | leu+ile+pro-oh=1357.44,leu+ile+pro-oh/phe=35.26,val=464.51 | -- | *DBT* | 248610 | c.1132c>t | hom | AR | - |  |
| Argininosuccinate aciduria | 1 | 98 | cit=83.8,ala/cit=3.9 | cit=120.32,ala/cit=2.06 | *ASL* | 608310 | c.331c>t | hom | AR | LP |  |
| Ornithine transcarbamylase deficiency | 1 | 99 | cit=5.34 | cit=7.56 | *OTC* | 300461 | c.829c>t  - | het | AR | - |  |
| **Organic acid metabolism** | **43** |  |  |  |  |  |  |  |  |  |  |
| 3- methyl Croton acyl coenzyme A carboxylase deficiency | 15 | 100 | c4dc+c5oh=1.98 | c4dc+c5oh=1.81 | *MCCC1* | 609010 | c.g295a  c.639+2t>a | het | AR | P  LP | M  F |
|  |  | 101 | c4dc+c5oh=8.64 | c4dc+c5oh=8.88 | *MCCC1* | 609010 | c.673c>t  c.161t>c | het | AR | P  P | F  M |
|  |  | 102 | c4dc+c5oh=0.99 | c4dc+c5oh=0.89 | *MCCC1* | 609010 | c.639+2t>a | hom | AR | P |  |
|  |  | 103 | c4dc+c5oh=3.04,c4dc+c5oh/c0=0.12,c4dc+c5oh/c8=50.67 | c4dc+c5oh=3.74,c4dc+c5oh/c0=0.17,c4dc+c5oh/c8=124.67 | *MCCC1* | 609010 | c.639+2t>a c.1614g>t | het | AR | P US |  |
|  |  | 104 | (c4dc+c5-oh)/c0=0.19,c4dc+c5-oh=3.47 | (c4dc+c5-oh)/c0=0.48,c4dc+c5-oh=5.82 | *MCCC1* | 609010 | c.639+2t>a  c.190g>a | het | AR | P  - |  |
|  |  | 105 | c4dc+c5-oh=7.33,(c4 dc+c5-oh)/c0=1.13 | c4dc+c5-oh=4.96,(c4dc+c5-oh)/c0=0.54 | *MCCC1* | 609010 | c.639+2t>a  - | het | AR | P |  |
|  |  | 106 | c4dc+c5-oh=1.24(c4dc+c5-oh)/c0=0.06 | c4dc+c5-oh=1.35,(c4dc+c5-oh)/c0=0.05 | *MCCC1* | 609010 | c.1069g>t  c.181g>t | het | AR | -  - |  |
|  |  | 107 | c4dc+c5-oh=5.98,(c4dc+c5-oh)/c0=0.24 | c4dc+c5-oh=16.79,(c4dc+c5-o h)/c0=1.15 | *MCCC1* | 609010 | c.1136g>a  c.388g>a | het | AR | -  - |  |
|  |  | 108 | c4dc+c5-oh=5.75,(c4dc+c5-oh)/c0=0 .64 | c4dc+c5-oh=7.35,(c4dc+c5- oh)/c0=0.9 | *MCCC2* | 609014 | c.1103delg  c.416c>t | het | AR | -  P |  |
|  |  | 109 | c4dc+c5-oh=5.77,(c4dc+c5-oh)/c0=0.31 | c4dc+c5-oh=9.26,(c4dc+c5-oh)/c0=1.97 | *MCCC1* | 609010 | c.863a>g | hom | AR | US |  |
|  |  | 110 | c4dc+c5-oh=3.1,(c4dc+c5-oh)/c0=0.14 | c4dc+c5-oh=2.71,(c4dc+c5-oh)/c0=0.14\| | *MCCC2* | 609014 | c.577c>t | hom | AR | -  LP |  |
|  |  | 111 | (c4dc+c5-oh)/c0=0.6,c4dc+c5-oh=8.87 | - | *MCCC2* | 609014 | c.592c>t  - | het | AR | - |  |
|  |  | 112 | (c4dc+c5-oh)/c0=0.23,c4dc+c5-oh=6.11 | - | *MCCC1* | 609010 | c.639+2t>a  c.872c>t | het | AR | P  US |  |
|  |  | 113 | c4d c+c5-oh=11.02,(c4dc+c5-oh)/c0=0.8 | c4dc+c5-oh=12.1,(c4dc+c5-oh)/c0=1.9 | *MCCC1* | 609010 | c.1679dupa  c.639+2t>a | het | AR | P  P |  |
|  |  | 114 | c4dc+c5oh=2.02,c4dc+c5oh/c0=0.15,c4dc+c5oh/c8=50.5 | c4dc+c5oh=2.33,c4dc+c5oh/c0=0.1,c4dc+c5oh/c8=116.5 | *MCCC1 MCCC2* | 609010/609014 | c.823g>a exon18 del c.80c>t | het | AR | US LP US P P |  |
| Glutaric academia 1 | 6 | 115 | c5dc+c6oh=3.9 | c5dc+c6oh=3.12 | *GCDH* | 608801 | c.1235c>a  c.1244-2a>c | het | AR | P  P | F  M |
|  |  | 116 | c5dc+c6oh=3.4 | c5dc+c6oh=3.93 | *GCDH* | 608801 | c.892g>a  c.261_506-433delinsata | het | AR | LP  LP | F  M |
|  |  | 117 | c5dc+c6oh=3.26 | c5dc+c6oh=3.19 | *GCDH* | 608801 | c.109_110delca  c.416c>g | het | AR | P  P | M  F |
|  |  | 118 | (c5dc+c6-oh)/(c4dc+c5-oh)=12.15,c5dc+c6-oh=1.58 | (c5dc+c6-oh)/(c4dc+c5-o h)=12.21,c5dc+c6-oh=1.71 | *GCDH* | 608801 | c.158c>g  c.554g>a | het | AR | -  - |  |
|  |  | 119 | (c5dc+c6-oh)/(c4dc+c5-oh)=14.8,c5dc+c6 -oh=2.96 | (c5dc+c6-oh)/(c4dc+c5-oh)=22.5,c5dc+c6-oh=3.6 | *GCDH* | 608801 | c.892g>a  c.1064g>a | het | AR | P  P |  |
|  |  | 120 | c5dc+c6-oh=3.61,(c5dc+c6- oh)/(c4dc+c5-oh)=13.885,(c5dc+c6-oh)/(c3dc+c4-oh)=72.2 | c5dc+c6-oh=2.18,(c5dc+c6-oh)/(c4dc+c5-oh)=9.909,(c5dc+c6- oh)/(c3dc+c4-oh)=43.6 | *GCDH* | 608801 | c.1240g>a  c.1186g>c | het | AR | P  - |  |
| Glutaric academia 2 | 1 | 121 | c4=1.87,c6=0.87,c8=1.42,c10=1.49,c12=2.30,c14=2.58,c14:1=1.64 | c4=054,c6=0.57,c8=0.44,c10=0.45,c12=0.33 | *ETFDH* | 231675 | c.872t>c | het | AR | - |  |
| Methylmalonic acidemia | 15 | 122 | c3=10.09 , c3/c2=0.92, c0=9.95, c2=9.95 | c3=5.82, c3/c2=3.23, c0=5.2, c2=1.8 | *MUT* | 609058 | c.1333c>g  c.1333-1g>a  c.729_730instt | het | AR | US  P  P |  |
|  |  | 123 | c3=6.7 c3/c2=0.51 | c3=6.64 c3/c2=0.91 | *MMACHC* | 609831 | c.1a>g  c.429+1g>t | het | AR | P  LP |  |
|  |  | 124 | c3=5.81,c3/c2=0.4 | c3=5.69,c3/c2=0.54 | *MMACHC* | 609831 | c.482g>a c.609g>a | het | AR | P P |  |
|  |  | 125 | c3=9.10,c3/c2=0.69 | c3=7.1,c3/c2=1.52 | *MMACHC* | 609831 | c.609g>a c.658_660del aag | het | AR | P P |  |
|  |  | 126 | c3=3.94,c3/c2=0.33 | c3=6.27,c3/c2=0.57 | *MMACHC* | 609831 | c.1a>g c.80a>g | het | AR | P P |  |
|  |  | 127 | c3/c2=0.27,c3=5.1 | c3/c2=0.47,c3=4.73 | *MUT* | 609058 | c.1663g>a  c.729_730instt | het | AR | LP  P |  |
|  |  | 128 | c3/c2=0.35 | c3/c2=0.46 | *MUT* | 609058 | c.729_730instt  c.1663g>a | het | AR | P  LP |  |
|  |  | 129 | c3/c2=0.39,c3=4.8 | c3/c2=0.38,c3=5.02 | *MUT* | 609058 | c.2131g>t  c.1663g>a | het | AR | -  LP |  |
|  |  | 130 | c3/c0=0.815,c3/c2=0.6 | - | *MUT* | 609058 | c.1677-1g>a  c.1280g>a | het | AR | P  P |  |
|  |  | 131 | c3/met=0.46,c3=5.49,c3/c2=0.66 | c3/c0=0.31,c3/met=0.68,c3=6.09,c3/c2=1.17 | *MMACHC* | 609831 | c.394c>t  c.609g>a | het | AR | P  P |  |
|  |  | 132 | c3/c0=0.38,c3/met=1.58,c3=6.8,c3/c2=0.58 | c3/c0=0.43,c3/met=0.94,c3=7.5,c3/c2=0.87, | *MMACHC* | 609831 | c.567dupt  c.609g>a | het | AR | P  P |  |
|  |  | 133 | c3/c2=0.66,c3/c0=0.41,c3/met=0.98 ,c3=6.18 | - | *MMACHC* | 609831 | c.658_660del | hom | AR | P |  |
|  |  | 134 | c3/c0=0.4,c3=4.91,c3/c2=0.75 | c3/c0=1.08,c3/met=0.42,c3=4.98, c3/c2=1.25 | *MUT* | 609058 | c.454c>t  c.2080c>t | het | AR | P  P |  |
|  |  | 135 | c0=7.98,c3/c2=0.16 | c3=2.07,c3/c2=0.29 | ND |  |  |  |  |  |  |
|  |  | 136 | c3/c2=0.98,c3=9.73 | - | ND |  |  |  |  |  |  |
| Isovalerate | 3 | 137 | c5=0.98,c5/c0=0.03,c5/c2=0.07,c5/c3=0.72 | c5=0.73,c5/c0=0.03,c5/c2=0.05,c5/c3=0.66 | *IVD* | 607036 | c.214g>a c.1051g>a | het | AR | P US | M F |
|  |  | 138 | c5/c0=2.52,c5=10.8 | c5=6.7,c5/c0=0.18 | *IVD* | 607036 | c.241c>t  - | het | AR | P  - |  |
|  |  | 139 | c5=1.97,c5/c0=0.07 | c5=1.94,c5/c0=0.05 | *IVD* | 607036 | c.1215a>g  c.466-2a>g | het | AR | -  LP |  |
| 2-methylbutylyl coenzyme A dehydrogenase deficiency | 1 | 140 | c5=0.51,c5/c0=0.03,c5/c2=0.04 | c5=0.99,c5/c0=0.05,c5/c2=0.2 | *ACADSB* | 600301 | c.416g>a c.848a>g | het | AR | US US |  |
| Ethyl malonic acidemia | 1 | 141 | c4=1.67,c4/c2=0.12,c4/c3=2.39,c5=0.56,c5/c0=0.04,c5/c2=0.04,c5/c3=0.8 | c4=1.14,c4/c2=0.15,c4/c3=2.92,c5=0.54,c5/c0=0.06,c5/c2=0.07,c5/c3=1.38 | *ETHE1* | 608451 | c.487c>t c.2t>a | het | AR | P LP | M F |
| Propionic acidemia | 1 | 142 | c3/met=0.52,c3=5.91,c3/c2=0.31 | c3/c2=0.38 | *PCCA* | 232000 | c.229c>t  c.2002g>a; | het | AR |  |  |
| **Fatty acid metabolism** | **52** |  |  |  |  |  |  |  |  |  |  |
| Carnitine uptake defect(CUD) | 18 | 143 | c0=6.55 | c0=4.31 | *SLC22A5* | 603377 | c.865c>t  c.1400c>g | het | AR | P  P | M  F |
|  |  | 144 | c0=2.88 | c0=3.09 | *SLC22A5* | 603377 | c.1433c>t c.428c>t | het | AR | P LP |  |
|  |  | 145 | c0=3.84,(c0+ c2+c3+c16+c18:1+c18)/cit=0.81 | c0=3.05 ,(c0+c2+c3+c16+c18:1+c18 )/cit=0.29 | *SLC22A5* | 603377 | c.1252c>t  - | het | AR | P |  |
|  |  | 146 | c0=5.9 | c0=5.91,(c0+ c2+c3+c16+c18:1+c18)/cit=0.58 | *SLC22A5* | 603377 | c.1400c>g | hom | AR | P |  |
|  |  | 147 | c0=5.21 | c0=6.08,(c0+c2+c3+c16+c18:1+c18)/cit=0.9, | *SLC22A5* | 603377 | c.1400c>g  c.51c>g | het | AR | P  LP |  |
|  |  | 148 | c0=7.75,c3=0.35 | c2=3.06,c3=0.19,c3dc+c4-oh=0.02,c4=0.08,c5=0.03,c8=0.01,c 16=0.29,c18=0.09,c18:1=0.27,c4/c3=0.42,leu+ile+pro-oh/phe =5.46,val/phe=4.64,orn/phe=5.74,c5dc+c6-oh=0.03\| | *SLC22A5* | 603377 | c.51c>g  c.1400c>g | het | AR | P  LP |  |
|  |  | 149 | c0=5.53,(c0+c2+c3+ c16+c18:1+c18)/cit=1.15 | c0=8.06,(c0+c2+c3+c16+c18:1+c18)/cit=0.6 | *SLC22A5* | 603377 | c.428c>t  c.51c>g | het | AR | -  P |  |
|  |  | 150 | c0=8.95,(c0+c2+c3+c16+c18:1+c18)/cit=1.15 | c0=3.32,(c0+c2+c3+c16+c18:1+c18)/cit=0.25 | *SLC22A5* | 603377 | c.760c>t  c.1400c>g | het | AR | P  LP |  |
|  |  | 151 | c0=3.11 ,(c0+c2+c3+c16+c18:1+c18)/cit=0.61 | phe /(c3+c16)=72.6,c0=4.44,(c0+c2+c3+c16+c18:1+c18 )/cit=0.5 | *SLC22A5* | 603377 | c.497+1g>t  c.652+1g>a | het | AR | LP  P |  |
|  |  | 152 | c0=6.67,(c0+c2+c3+c16+c18:1+c18)/cit= 1.076 | c0=5.75,p he/(c3+c16)=60.071,(c0+c2+c3+c16+c18:1+c18)/cit=0.515 | *SLC22A5* | 603377 | c.394-1g>t  c.1400c>g | het | AR | LP  LP |  |
|  |  | 153 | c0=4.28,(c0+c2+c3+c16+c18 :1+c18)/cit=0.924 | c0=5.45,phe/(c3+c16)=141.829,(c0+c2+c3+c16+c18:1+c18)/c it=0.466 | *SLC22A5* | 603377 | c.760c>t  c.1400c>g | het | AR | P  LP |  |
|  |  | 154 | c0=4.61(c0+c2+c3+c16+c18:1+c1 8)/cit=0.85 | c0=7.39,phe/(c3+c16)=86.4,(c0+c2+c3+c16+c18:1+c18)/cit=0.66 | *SLC22A5* | 603377 | c.497+1g>t  c.1462c>t | het | AR | LP  P |  |
|  |  | 155 | c0=7.44,phe/(c3+c16)=65.01,(c0 +c2+c3+c16+c18:1+c18)/cit=0.61 | phe/(c3+c16)=71.51,(c0 +c2+c3+c16+c18:1+c18)/cit=1.01 | *SLC22A5* | 603377 | c.1400c>g | hom | AR | LP |  |
|  |  | 156 | c0=3.28,(c0+c2+c3+c16+c18:1+c18)/cit=0.83 | phe/(c3+c16)=308.5,(c0+c2+c 3+c16+c18:1+c18)/cit=0.2,c0=2.37 | *SLC22A5* | 603377 | c.1433c>t | hom | AR | P |  |
|  |  | 157 | c0=6.18 | phe/(c3+c16)=75.02,(c0+c2+c3+c16+c18:1+c18)/cit=0.57,c0=7.19 | *SLC22A5* | 603377 | c.51c>g  c.1400c>g | het | AR | P  LP |  |
|  |  | 158 | c0=8.12 | c0=5.6,phe/(c3+c16)=137.933,(c0+c2+c3+c16+c18:1+c18)/cit=0.513 | *SLC22A5* | 603377 | c.760c>t  c.1400c>g | het | AR | P  LP |  |
|  |  | 159 | c0=3.84,(c0+ c2+c3+c16+c18:1+c18)/cit=0.81 | c0=3.05 ,(c0+c2+c3+c16+c18:1+c18 )/cit=0.29 | *SLC22A5* | 603377 | c.1252c>t  - | het | AR | P |  |
|  |  | 160 | c0=5.9 | c0=5.91,(c0+ c2+c3+c16+c18:1+c18)/cit=0.58 | *SLC22A5* | 603377 | c.1400c>g | hom | AR | LP |  |
| Medium chain acyl coenzyme A dehydrogenase deficiency | 7 | 161 | c6=0.72，c6dc=0.31，c8=0.49，  c10=0.34，c10：1=0.47 | c6=0.37，c6dc=0.2，c8=1.2  c10:1=0.17 | *ACADM* | 607008 | c.799g>a  c.1085g>a | het | AR | P  P | F  M |
|  |  | 162 | c6=0.33,c8=2.37,c10=0.24,c10:1=0.34 | c6=0.52,c8=3.16,c10=0.24,c10:1=0.56 | *ACADM* | 607008 | c.449_452del | hom | AR | P | F M |
|  |  | 163 | c6=0.24,c8=1.38,c10:1=0.37,c8/c2=0.17,c8/c10=12.55 | c6=0.44,c8=2.45,c10:1=0.45,c8/c2=0.41,c8/c10=15.31 | *ACADM* | 607008 | c.1085g>a | hom | AR | P | F M |
|  |  | 164 | c6=0.92,c8=14.52,(c4dc+c5-oh)/c8=0 .01,c8/c2=0.7 | c6=0.68,c8=3.44,(c4dc+c5-oh)/c8=0.05 c8/c2=0.42 | *ACADM* | 607008 | c.790g>t | het | AR | p |  |
|  |  | 165 | c6=0.47,c8=1.18,(c4dc+c5-oh)/c8=0.13,c8/c2=0.09 | c6=0.52,c8=1.36,(c4dc+c5-oh)/c8=0.18,c8/c2=0.17 | *ACADM* | 607008 | c.970g>a  c.1238g>a | het | AR | -  US |  |
|  |  | 166 | c8/c2=0.06,c6=0.55,c8=1.99,(c4dc+c5-oh)/c8=0.08 | c8/c2=0.02,c6=0.17,c8=0.27,(c4dc+c5-oh)/c8=0. 78 | *ACADM* | 607008 | c.449_452delctga  c.1248t>g | het | AR | P  - |  |
|  |  | 167 | c6=0.09 | c6=0.13,c8=0.22,(c4dc+c5-oh)/c8=0.86 ,c8/c2=0.02 | *ACADM* | 607008 | c.499_452delctga  c.589a>g | het | AR | -  - |  |
| Short chain acyl coenzyme A dehydrogenase deficiency | 20 | 168 | c4=1.15 | c4=1.1 | *ACADS* | 606885 | c.164c>t  c.1031a>g | het | AR | P  P | - |
|  |  | 169 | c4=1.09 | c4=1.28 | *ACADS* | 606885 | c.1031a>g  c.1130c>t | het | AR | P  P | - |
|  |  | 170 | c4=1.08,(c3dc+c4oh)/c4=0.1,c4/c2=0.09,c4/c3=1.06 | c4=1.41,(c3dc+c4oh)/c4=0.06,c4/c2=0.16,c4/c3=1.78 | *ACADS* | 606885 | c.1031a>g c.164c>t | het | AR | P P |  |
|  |  | 171 | c4=0.53 | c4=0.43 | *ACADS* | 606885 | c.1031a>g c.1130c>t | het | AR | P LP |  |
|  |  | 172 | c4=1.50,c4/c2=0.09,c4/c3=1.06 | c4=1.09,c4/c2=0.13,c4/c3=2.1 | *ACADS* | 606885 | c.172c>t c.617g>a | het | AR | LP US |  |
|  |  | 173 | c4=1.21,c4/c2=0.06,c4/c3=0.42 | c4=1.8,c4/c2=0.11,c4/c3=0.94 | *ACADS* | 606885 | c.536t>c  - | het | AR | US |  |
|  |  | 174 | c4=0.70,c4/c2=0.06,c4/c3=0.57 | c4=0.70,c4/c2=0.09,c4/c3=0.64 | *ACADS* | 606885 | c.164c>t c.1130c>t | het | AR | P P | F M |
|  |  | 174 | c4=0.64,c4/c2=0.04,c4/c3=0.49 | c4=1.14,c4/c2=0.11,c4/c3=1.05 | *ACADS* | 606885 | c.164c>t c.1000c>t c.444g>t | het | AR | P LP US | M F M |
|  |  | 176 | c4=1.95,c4/c2=0.08,c4/c3=1.34 | c4=1.66,c4/c2=0.14,c4/c3=2.31 | *ACADS* | 606885 | c.1031a>g | hom | AR | P | M |
|  |  | 177 | c4=0.89,c4/c2=0.18 | c4=0.91,c4/c2=0.09 | *ACADS* | 606885 | c.737g>a | hom | AR | - |  |
|  |  | 178 | c4=0.8,c4/c2=0.1 | c4=1.42,c4/c2=0.06 | ND |  |  |  |  |  |  |
|  |  | 179 | c4=1.47,c4/c2=0.09 | c4=1.18,c4/c2=0.17 | *ACADS* | 606885 | c.1031a>g  c.1055c>t | het | AR | LP  - |  |
|  |  | 180 | c4=1.08,c4/c2=0.14 | c4=0.98,c4/c2=0.12 | *ACADS* | 606885 | c.1031a>g  c.1130c>t; | het | AR | LP  - |  |
|  |  | 181 | c4/c2=0.079,c4=2.03 | c4=1.78,c4/c2=0.078 | *ACADS* | 606885 | c.1031a>g  c.1054g>a | het | AR | LP  US |  |
|  |  | 182 | c4=2.01,c4/c2=0.076 | c4=1.38,c4/c2=0.177 | *ACADS* | 606885 | c.1031a>g  c.322g>a | het | AR | LP  LP |  |
|  |  | 183 | c4=1.43,c4/c2=0.116 | c4=1.58,c4/c2=0.204 | *ACADS* | 606885 | c.164c>t  c.1031a>g | het | AR | LP  LP |  |
|  |  | 184 | c4=0.51,c4/c2=0.04 | c4=0.54,c4/c2=0.06 | *ACADS* | 606885 | c.164c>t  c.1130c>t | het | AR | LP  - |  |
|  |  | 185 | c4=1.1,c4/c2=0.13 | c4=1.3,c4/c2=0.17 | *ACADS* | 606885 | c.973c>t  c.1031a>g | het | AR | -  LP |  |
|  |  | 186 | c4/c2=0.09,c4=1.35 | c4/c2=0.17,c4=1.07 | *ACADS* | 606885 | c.164c>t  c.1031a>g | het | AR | LP  LP |  |
|  |  | 187 | c4=0.88,c4/c2=0.052 | c4=1.19,c4/c2=0.15 | *ACADS* | 606885 | c.1031a>g  c.1130c>t | het | AR | LP  - |  |
| Very long chain acyl coenzyme A dehydrogenase deficiency | 5 | 188 | c14:1/c2=0.55,c14:1/c16=0.6 2,c14:1=1.51 | c14:1/c2=0.61,c14:1/c16=0.71,c14:1=1.95 | *ACADVL* | 609575 | c.887_888delct | hom | AR | - |  |
|  |  | 189 | c14:1/c2=0.51,c14:1/c16=0.6 | c14 :1=1.95 | *ACADVL* | 609575 | c.1280g>a  c.1345g>c | het | AR | LP  US |  |
|  |  | 190 | c14:1/c 16=0.5,c14:1/c2=0.25,c14:1=4.27 | - | *ACADVL* | 609575 | c.1349g>a  c.895>g~~;~~ | het | AR | LP  -  LP |  |
|  |  | 191 | c14:1/c16=0.53,c14:1/c2=0.06,c14:1=0.63 | c14:1/c16=1.15,c14: 1/c2=0.16,c14:1=1.42 | *ACADVL* | 609575 | c.553g>a  c.848t>c | het | AR | LP  P |  |
|  |  | 192 | c14:1/c16=1.129, c14:1/c2=0.331,c14:1=2.1 | c14:1/c16=1.386,c 14:1/c2=0.553,c14:1=2.91 | *ACADVL* | 609575 | c.642_643delct  c.1349g>a | het | AR | -  LP |  |
| ẞ-ketothiolase deficiercy | 2 | 193 | c3dc+c4oh=3.58，c4dc+c5oh=1.31  c5：1=0.31 | c3dc+c4oh=1.07，c4dc+c5oh=1.01  c5：1=0.24 | *ACAT1* | 607809 | c.721dupa  c.928g>c | het | AR | LP  US | F  M |
|  |  | 194 | c3dc+c4oh=1.22,c4dc+c5oh=0.71,c5:1=0.20 | c3dc+c4oh=0.42,c4dc+c5oh=0.88,c5:1=0.39 | *ACAT1* | 607809 | c.238+1g>a c.1163g>t | het | AR | P US |  |
| **Total** | 194 |  |  |  |  |  |  |  |  |  |  |

Note:

ND: Not done.

P: Pathogenic. LP: Likely pathogenic. US:Uncertain significance.

AR: Autosomal recessive inheritance. AD: Autosomal dominant inheritance.

het: heterozygotes. hom: homozygosis.

F: Father. M: Mother
